# Integrated analysis of miRNA and mRNA expression profiles in testes of Duroc and Meishan boars

**DOI:** 10.1186/s12864-020-07096-7

**Published:** 2020-10-02

**Authors:** Haisheng Ding, Min Liu, Changfan Zhou, Xiangbin You, Tao Su, Youbing Yang, Dequan Xu

**Affiliations:** 1 Key Laboratory of Swine Genetics and Breeding of Ministry of Agriculture and Rural Affairs, and Key Lab of Agricultural Animal Genetics, Breeding and Reproduction of Ministry of Education, Huazhong Agricultural University, Wuhan, 430070 People’s Republic of China; 2 Anhui Key Laboratory of Livestock and Poultry Product Safety Engineering, Institute of Animal Husbandry and Veterinary Medicine, Anhui Academy of Agricultural Sciences, Hefei, 230031 People’s Republic of China; 3 College of Animal Science and Technology, Henan University of Science & Technology, Luoyang, 471023 People’s Republic of China

**Keywords:** Meishan boar, Duroc boar, Testis, Sexual development, miRNA, Integrating analysis

## Abstract

**Background:**

MicroRNAs (miRNAs) are small non-coding RNAs playing vital roles in regulating posttranscriptional gene expression. Elucidating the expression regulation of miRNAs underlying pig testis development will contribute to a better understanding of boar fertility and spermatogenesis.

**Results:**

In this study, miRNA expression profile was investigated in testes of Duroc and Meishan boars at 20, 75, and 270 days of age by high-throughput sequencing. Forty-five differentially expressed miRNAs were identified from testes of Duroc and Meishan boars before and after puberty. Integrated analysis of miRNA and mRNA profiles predicted many miRNA-mRNA pairs. Gene ontology and biological pathway analyses revealed that predicted target genes of ssc-mir-423-5p, ssc-mir-34c, ssc-mir-107, ssc-mir-196b-5p, ssc-mir-92a, ssc-mir-320, ssc-mir-10a-5p, and ssc-mir-181b were involved in sexual reproduction, male gamete generation, and spermatogenesis, and GnRH, Wnt, and MAPK signaling pathway. Four significantly differentially expressed miRNAs and their predicted target genes were validated by quantitative real-time polymerase chain reaction, and phospholipase C beta 1 (*PLCβ1)* gene was verified to be a target of ssc-mir-423-5p.

**Conclusions:**

This study provides an insight into the functional roles of miRNAs in testis development and spermatogenesis and offers useful resources for understanding differences in sexual function development caused by the change in miRNAs expression between Duroc and Meishan boars.

## Background

Testis is an important male reproductive and endocrine organ which is a critical tissue for spermatogenesis. Spermatogenesis is a complicated process including mitotic cell division, meiosis, and the process of spermiogenesis [1]. Spermatogenesis is strictly regulated by the expression of stage-specific genes in testis at both transcription and post-transcription levels [2]. Identifying key regulators in testis development and spermatogenesis will provide valuable insights into the mechanism of sexual function development [3].

As a class of endogenous small (~ 22 nucleotides) noncoding RNAs, miRNAs mediate post-transcriptional gene expression in animals and fine-tune the expression of approximately 30% of all mammalian protein-coding genes [4–6]. In addition, miRNAs regulate gene expressions not only at post-transcriptional levels but also at the transcriptional level by RNA-RNA interactions [7]. A large number of miRNAs were found to have been involved in many biological processes including cell growth and differentiation, embryo development and sperm morphology and mobility [8, 9]. miRNA expression patterns were significantly different between immature and mature mouse testes and miR-449a/b and miR-34b/c function redundantly in the regulation of male germ cell development [10, 11]. Comparative profiling of miRNAs expressed in the newborn, young adult, and aged human epididymides showed that 127 miRNAs were exclusively or preferentially expressed in the newborn epididymis, but only 3 and 2 miRNAs abundantly expressed in the adult and aged epididymides, respectively [12]. miRNAs were also involved in spermatogenesis, and their presence or absence in mature sperm was highly related to aberrant development, function and/or fertility [8, 13, 14]. Adult porcine miRNAs in ovary and testis have been identified and co-expression patterns of X-linked miRNAs in adult porcine gonads were found [15]. miRNA expression patterns between sexually immature (60-day) and mature (180-day) pig testes also have been evaluated and indicated that miRNAs had an important role in regulating spermatogenesis [16]. However, little has been reported about miRNA expression in the testes at various stages of development across pig breeds.

One of our previous studies of testis transcriptional profile revealed numerous differentially expressed genes (DEGs) and important biological pathways were significantly correlated to mammalian reproduction between Meishan and Duroc boars at 20, 75, and 270 days of age [17]. It is interesting to further investigate how miRNAs are involved in regulating sexual function development by fine-turning gene expression [18]. Chinese Meishan pigs are one of the most prolific pig breeds in the world and reach puberty at a relatively younger age (56–84 days) than conventional boars (120–180 days) [19]. Duroc sires are utilized most frequently as a Terminal/Paternal sire in a terminal cross-breeding programe. Our previous work also showed spermatogenesis occurred prior to 75 days in Meishan boars and their spermatogenesis came earlier than Duroc boars, but the number of spermatogonia and Sertoli cells in Meishan boars are less than that in Duroc boars at adulthood [17]. Testis size in Chinese Meishan boars is only half that of conventional boars at maturity. In addition, Meishan boars accumulate Sertoli cells and seminiferous tubules at a more rapid rate compared with white composite boar during the first month after birth [17, 20, 21]. The diameter and number of seminiferous tubules determine the onset of puberty in males [17]. The physiological attributes mentioned above of Chinese Meishan boars make them highly prolific, which render them a valuable animal model for examining the mechanism of sexual function development and sperm production of boars.

Based on miRNA-mRNA pairwise correlations and computational target prediction of miRNA, the miRNA and mRNA expression profiles were integrated to construct miRNA-mRNA regulatory networks which could potentially affect testicular development and spermatogenesis. This study revealed a large number of miRNAs that potentially regulates pig testis development and spermatogenesis and provides a better understanding of differences in sexual function between Meishan and Duroc boars.

## Results

### Overview of small RNA libraries

**Fig. 1:**
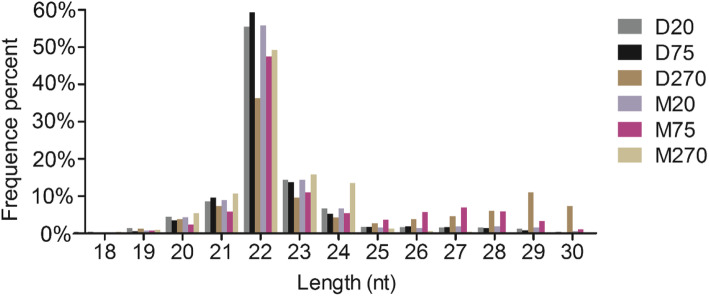
Length distribution and abundance of the small RNA libraries

In order to identify differentially expressed miRNAs during the process of testicular development of Duroc and Meishan boars, six small RNA (sRNA) libraries of testis tissues of 20-, 75-, 270-day-old Duroc and Meishan boars (D20, D75, D270, M20, M75, and M270) were constructed and sequenced by the Illumina HiSeq™ 2000 platform. In total, 13,335,120, 13,051,493, 13,352,724, 13,606,755, 12,695,970, and 13,721,075 raw reads were generated in D20, D75, D270, M20, M75, and M270, respectively. After removing the low-quality sequences and adaptors, and then discarding the sequences shorter than 18 nt, 13,133,806, 12,892,394, 13,167,240, 13,441,877, 12,561,392, and 13,522,792 clean reads were obtained and used for further analysis (Table 1). A total of 1,078,105 and 878,097 unique sRNA from Duroc and Meishan boar testes were mapped to the porcine reference genome (Sscrofa10.2), respectively (Table 2). Read length distribution analyses of the six sRNA libraries showed that the dominant length of sRNAs was 22 nt, accounting for at least 36.24% of the population (Fig. 1). More 22 nt sRNAs were found in D20, D75, and M20 than in D270, M75, and M270. While few sRNAs with the length of 18 to 19 nt and 25 to 30 nt were detected, and sRNAs with the length of 25 to 30 nt may mainly represent Piwi-interacting RNAs (piRNA). These results are similar to those of previous study of pig [22].

**Table 1:** Quality analyses of small RNA-seq data

Sample	Total reads	high_quality	Smaller_than_18nt	PolyA	Clean reads
D20	13,335,120	13,268,836 (100%)	79,018 (0.60%)	15	13,133,806 (98.98%)
D75	13,051,493	12,987,508 (100%)	41,877 (0.32%)	21	12,892,394 (99.27%)
D270	13,352,724	13,289,346 (100%)	70,860 (0.53%)	16	13,167,240 (99.08%)
M20	13,606,755	13,537,863 (100%)	42,915 (0.32%)	22	13,441,877 (99.29%)
M75	12,695,970	12,642,443 (100%)	23,239 (0.18%)	28	12,561,392 (99.36%)
M270	13,721,075	13,658,694 (100%)	83,811 (0.61%)	6	13,522,792 (99.01%)

**Table 2:** The reads mapping to reference genome from small RNA-seq data

Sample	Unique sRNAs		Total sRNAs	
	Total	mapped	Total	mapped
D20	402,022	269,359 (67%)	13,133,806	10,689,788 (81.39%)
D75	433,787	293,153 (67.58%)	12,892,394	10,942,820 (84.88%)
D270	787,613	515,593 (64.46%)	13,167,240	10,849,401 (82.4%)
M20	417,327	269,558 (64.59%)	13,441,877	10,828,754 (80.56%)
M75	857,893	554,629 (64.65%)	12,561,392	10,279,770 (81.84%)
M270	117,259	53,910 (45.98%)	13,522,792	10,279,770 (81.84%)

### Differentially expressed miRNAs across Duroc and Meishan boars

The expression profiles of known miRNAs from the six samples were analyzed and 36–139 significantly differentially expressed miRNAs (DE miRNAs) (*P* ≤ 0.05, |log2^Ratio^| ≥ 1) were filtered in each pairwise comparison (Table 3; Additional file 9: Table S6). For example, the comparisons between the two breeds indicated that the number of DE miRNAs in the 75- and 270- day time points were substantially larger than that in the 20- day time point. These results suggested that significant differences existed during testicular development between Duroc and Meishan boars at the age of 75 and 270 days. More significantly DE miRNAs were detected in Meishan boars at age of 20 to 75 days than those in Duroc boars, which suggested that the testis developed at a faster rate in Meishan boars than in Duroc boars from 20 to 75 days. These findings were consistent with those in previous study of mRNA expression data of matched samples [17].

**Table 3:** Analysis of differentially expressed miRNAs

Sample	Total	Up regulated miRNA	Down regulated miRNA
D20-vs-M20	39	22	17
D75-vs-M75	100	21	79
D270-vs-M270	120	104	16
D20-vs-D75	36	13	23
D20-vs-D270	138	19	119
D75-vs-D270	139	22	117
M20-vs-M75	111	16	95
M20-vs-M270	125	64	61
M75-vs-M270	116	93	23

**Fig. 2:**
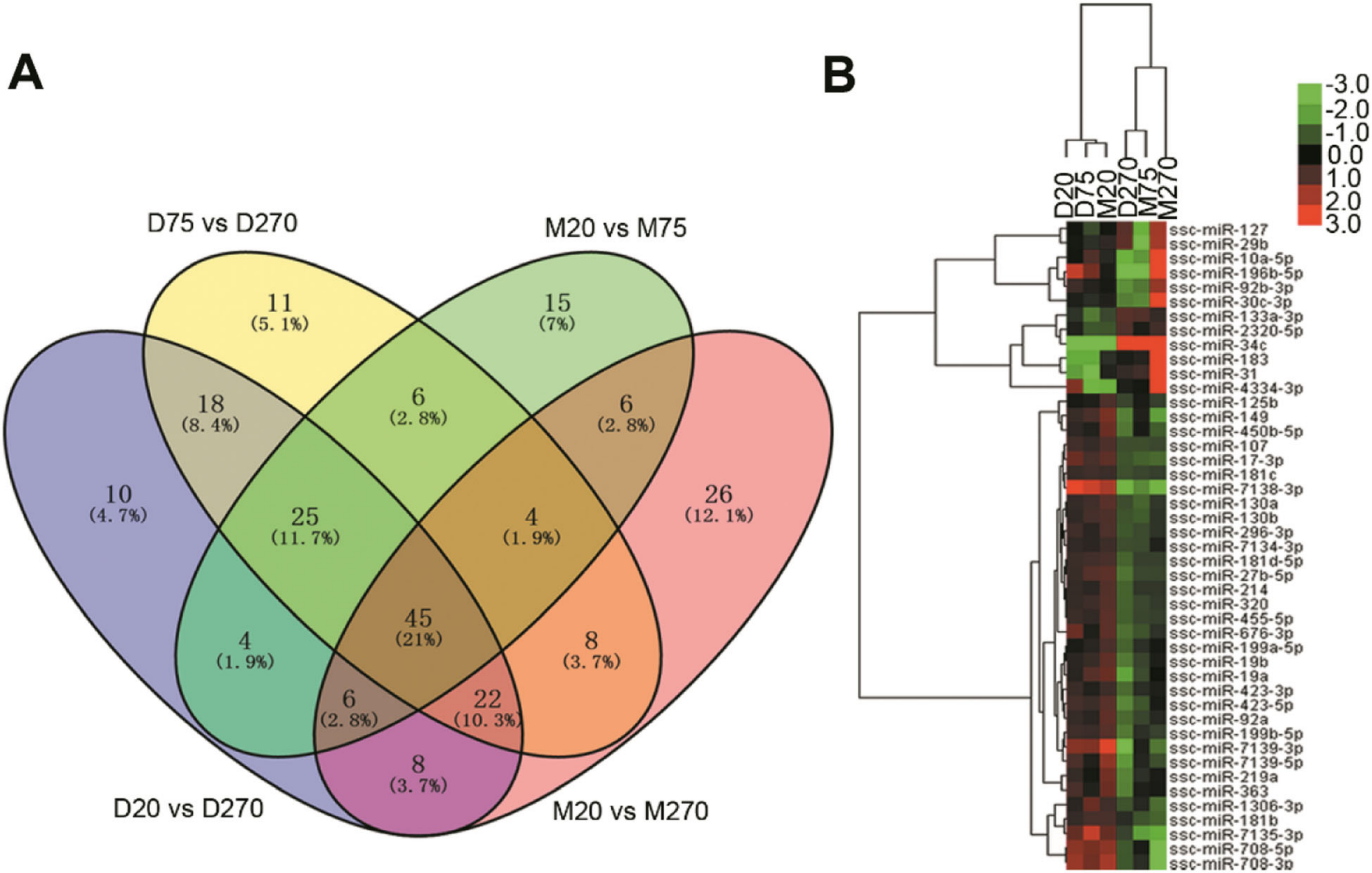
Analysis of differentially expressed miRNAs. **a** Venn diagram of differentially expressed miRNAs from D20 vs D270, D75 vs D270, M20 vs M75 and M20 vs M270 groups. The number of genes is given in the middle of each figure section. **b** The heatmap of the subset miRNAs from intersection of the Venn diagram

Venn diagram showed 45 significantly DE miRNAs were filtered from the four pairwise comparisons (D20 vs D270, D75 vs D270, M20 vs M75, and M20 vs M270) (Fig. 2a). The four pairs represented the comparisons before and after puberty since D270, M75, and M270 have reached puberty. Figure 2b showed two main sample branches (D20, D75, and M20 versus D270, M75, and M270), which indicated that the expression pattern of M20 was similar to those of D20 and D75, and that the expression pattern of D270 was similar to those of M75 and M270. The results were consistent with those of previous study of expression pattern of mRNA [17], demonstrating large differences existed in the process of testicular development between Meishan and Duroc boars. It could be concluded that miRNAs were pivotal factors regulating sexual function development.

### Integrated analysis between differentially expressed miRNAs and target mRNAs in Duroc and Meishan boars at different stages

The mRNA expression data of six samples from our previous study were used for a pairwise integrated analysis [17]. Through the Trinity de novo assembly method, 20,525 non-redundant genes were obtained from the six samples (Additional file 1: Table S1), 19,310 (94.08%) and 18,241(88.87%) genes were mapped against Kyoto Encyclopedia of Genes and Genomes (KEGG) and (Gene Ontology) GO databases, respectively (Additional file 2: Table S2).

**Fig. 3:**
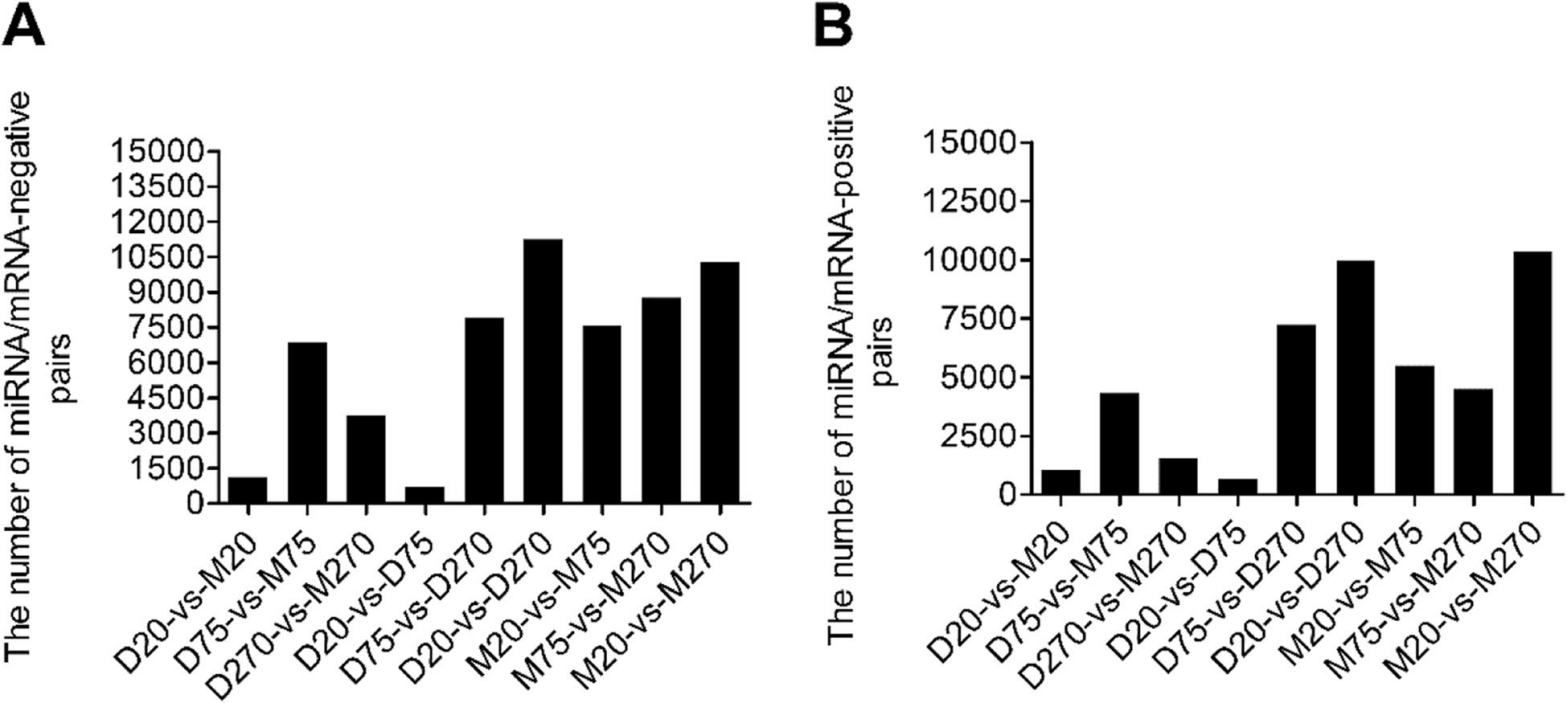
The statistics for miRNA/mRNA-regulation pairs in Duroc and Meishan boars. **a** The number of miRNA/mRNA-negative pairs. **b** The number of miRNA/mRNA-positive pairs

In order to better understand the potential roles of the miRNAs during the process of testicular development, computational target prediction was performed using Targetscan and miRanda. Then we performed integrated analyses of differentially expressed miRNAs and target mRNAs at the expression levels. A large number of correlated miRNA-mRNA pairs were detected in each pairwise comparisons (Fig. 3). The number of miRNA/mRNA-negative pairs between Duroc and Meishan boars at 75-day time point was obviously higher than that at 20- and 270-day time points, and more negative pairs were detected from 20 to 75 days in Meishan boars than those in Duroc boars (Fig. 3a). The previous study demonstrated that Meishan boars attained puberty and their testes generated sperms prior to 75 days earlier than Duroc boars [17]. These findings indicate that miRNAs as negative gene expression regulators significantly control the expression of genes involved in regulating the process of testicular development. Meanwhile, the number of miRNA/mRNA-positive pairs was also compared between Duroc and Meishan boars (Fig. 3b) with a similar tendency found in Duroc and Meishan boars at different ages. These results reveal that the subset of miRNAs may function as enhancers activating the transcription of genes which play an important role during the process of testicular development.

**Fig. 4:**
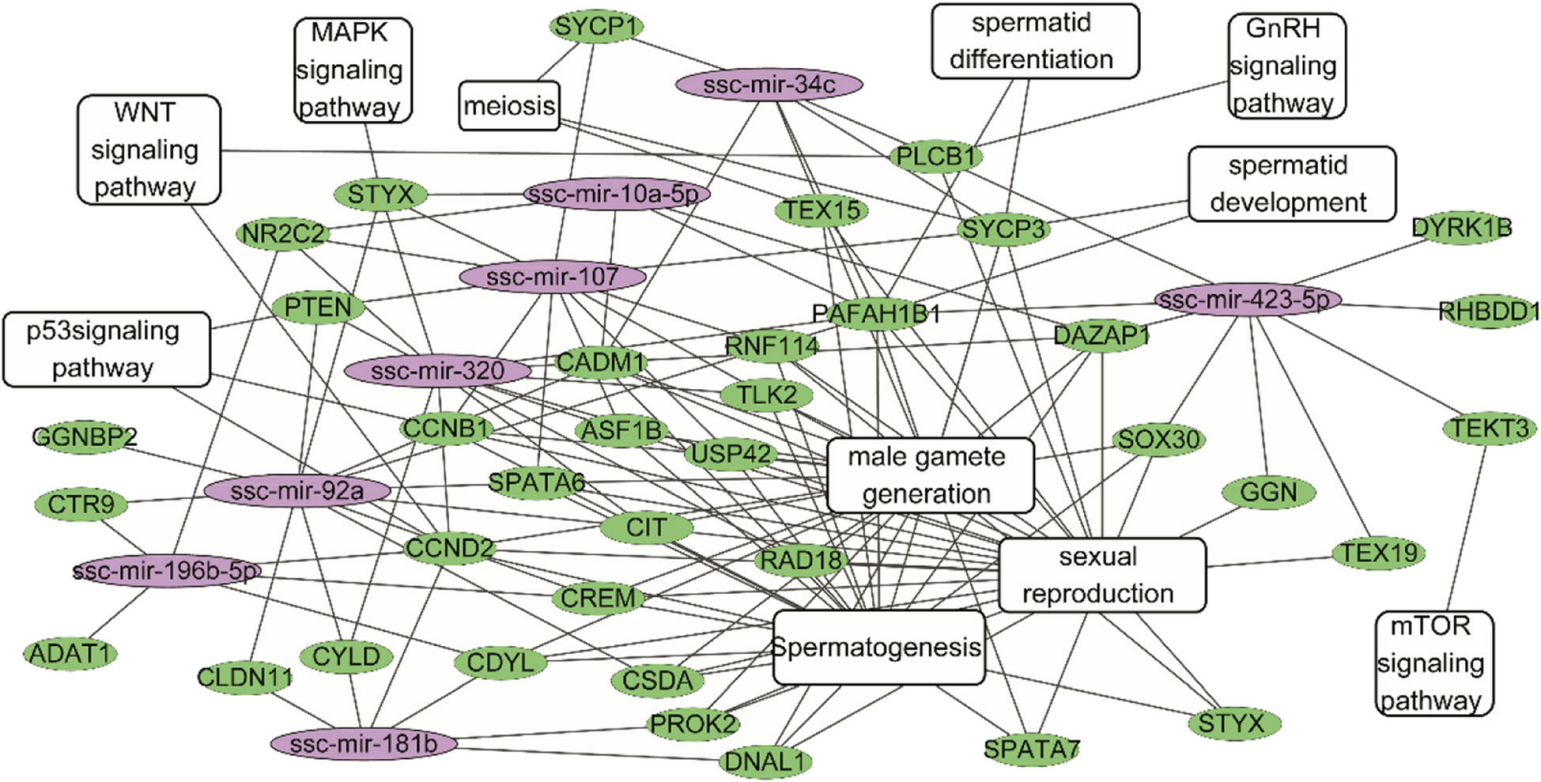
Differentially expressed miRNA-mRNA pairs and regulatory network between Duroc and Meishan boars. Purple indicates miRNAs, green indicates genes, white boxes indicate GO terms and KEGG pathways mapped by genes. GO, Gene Ontology; KEGG, Kyoto Encyclopedia of Genes and Genomes

A representative miRNA-mRNA regulatory network of biological pathways was shown in Fig. 4. Eight DE miRNAs (ssc-mir-423-5p, ssc-mir-34c, ssc-mir-107, ssc-mir-196b-5p, ssc-mir-92a, ssc-mir-320, ssc-mir-10a-5p, and ssc-mir-181b) were selected from 45 miRNAs deriving from Fig. 2 serving as functional miRNAs in testicular development of Meishan and Duroc boars according to their annotations and the potential relationship between miRNAs and spermatogenesis and gonad development. GO analysis and KEGG functional annotation of potential target genes of eight DE miRNAs were performed to detect the functional characteristics of miRNAs. A large number of target genes were assigned to the functional categories related to sexual reproduction, male gamete generation, spermatogenesis, sperm development as well as meiosis, indicating that the eight miRNAs were highly involved in spermatogenesis and testis development. Five important pathways including GnRH, Wnt, p53, mTOR, and MAPK signaling pathway related to the regulation of male sexual function were enriched by functional genes including phospholipase C beta 1 (*PLCβ1*) involved in GnRH and Wnt signaling pathway with *PLCβ1* being the target of ssc-mir-423-5p and ssc-mir-34c, serine/threonine/tyrosine interacting protein (*STYX*) involved in MAPK signaling pathway with *STYX* being the target of ssc-mir-320, ssc-mir-10a-5p, ssc-mir-92a and ssc-mir-107; cyclin D2 (*CCND2*), phosphatase and tensin homolog (*PTEN*), and cyclin B1 (*CCNB1*) involved in the p53 signaling pathway with the 3 genes being the target of ssc-mir-320, and so on. The representative miRNA-mRNA regulatory networks contained biological pathways regulating male sexual function, which illustrated a complex relationship and interaction between the two biomolecular types.

### Verification of DE miRNAs and target verification of ssc-mir-423-5p

**Fig. 5:**
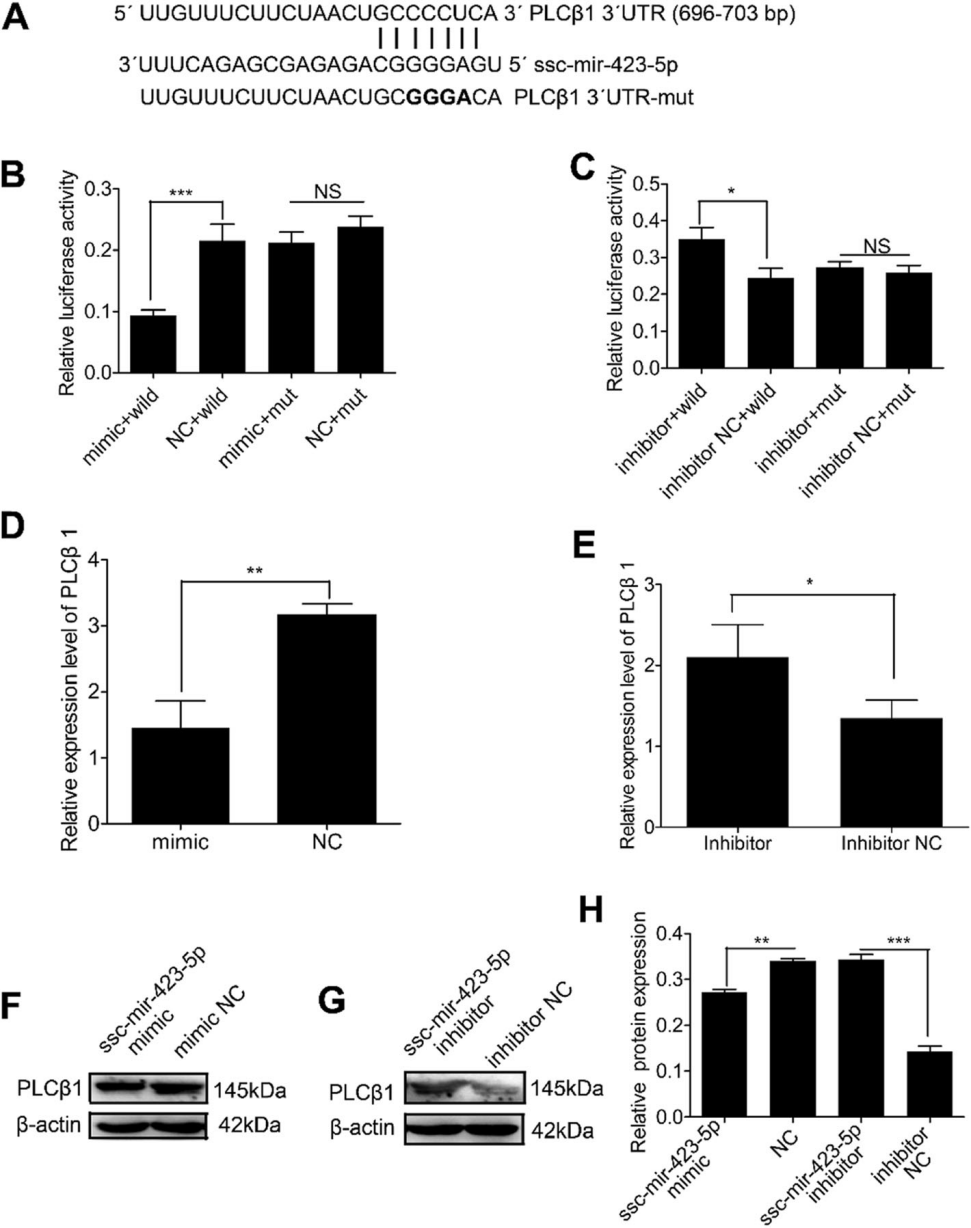
Identification of *PLCβ1* as a direct target of ssc-mir-423-5p in ST cells. **a** Binding sites for ssc-mir-423-5p in the 3ˈ-UTR of *PLCβ1* predicted by TargetScan. Bold font indicate sequences that were mutated to abolish the interaction between ssc-mir-423-5p and *PLCβ1* 3’UTR. **b** Luciferase activity was analyzed after co-transfecting pmirGLO-PLCβ1–3′-UTR or pmirGLO-PLCβ1–3′-UTR -mut and ssc-mir-423-5p mimic or mimic NC into ST cells at 24 h. **c** Luciferase activity was analyzed after co-transfecting pmirGLO-PLCβ1–3′-UTR or pmirGLO-PLCβ1–3′-UTR -mut and ssc-mir-423-5p inhibitor or inhibitor NC into ST cells at 24 h. **d** *PLCβ1* mRNA levels were detected at 48 h after swine cells were transfected with ssc-mir-423-5p mimic or mimic NC. **e** *PLCβ1* mRNA levels were detected at 48 h after swine cells were transfected with ssc-mir-423-5p inhibitor or inhibitor NC. **f** Western blotting analysis was used to detect PLCβ1 protein expression levels at 72 h after ST cells were transfected with ssc-mir-423-5p mimic and mimic NC. **g** Western blotting analysis was used to detect PLCβ1 protein expression levels at 72 h after ST cells were transfected with ssc-mir-423-5p inhibitor and inhibitor NC. **h** The quantification of PLCβ1 protein. *P < 0.05, **P < 0.01, ***P < 0.001, *PLCβ1*, phospholipase C beta 1; UTR, Untranslated Region; ST, swine testis; NC, negative control; N.S., nonsignificant

To evaluate our DE miRNAs library, the expression profiles of 3 DE miRNAs (ssc-mir-181b, ssc-mir-423-5p, ssc-mir-196b-5p), four DEGs from the miRNA-mRNA interaction networks, and ssc-mir-4334-3p from the Venn diagram in Fig. 2a, all of which were highly related to boar sexual function and reproduction, were further analyzed by quantitative real-time PCR (qRT-PCR) with specific primers. As shown in Additional file 3: Figure S1, the results of RNA-seq data and qRT-PCR data were identical. *CYLD* did not show consistent expression between RNA-seq and qRT-PCR data from Duroc and Meishan boars at age of 20 and 270 days, which was probably caused by the sensitivity of the different methods. In general, the results of qRT-PCR validated the RNA-seq results and demonstrated the reliability of our data.

ssc-mir-423-5p was one of the differentially expressed miRNAs and was selected as a candidate miRNA for analyzing male sexual function. *PLCβ1* was predicted to be a target of ssc-mir-423-5p (Fig. 5a). The dual-luciferase reporter assay system analyzed the interaction between ssc-mir-423-5p and *PLCβ1* gene. The analysis results indicated that luciferase activity was significantly suppressed after we co-transfected ssc-mir-423-5p mimic (Additional file 4: Table S3) and pmirGLO- PLCβ1–3′-UTR. However, luciferase activity was not significantly changed when we co-transfected ssc-mir-423-5p mimic and pmirGLO- PLCβ1–3′-UTR -mut into Swine Testis (ST) cells (Fig. 5b). Meanwhile, ssc-mir-423-5p inhibitor significantly promoted luciferase activity after we co-transfected ssc-mir-423-5p inhibitor and pmirGLO- PLCβ1–3′-UTR, and luciferase activity was unchanged after we co-transfected ssc-mir-423-5p inhibitor and pmirGLO-PLCβ1–3′-UTR-mut into ST cells (Fig. 5c). qRT-PCR and western blotting analyses revealed that PLCβ1 mRNA and protein expression levels were significantly reduced after ssc-mir-423-5p mimic was transfected into ST cells, whereas the inhibition of ssc-mir-423-5p increased the expression of *PLCβ1* mRNA and protein in ST cells (Fig. 5d-g, Additional file 5: Figure S2, Additional file 6: Figure S3). These results suggested that *PLCβ1* was the target gene of ssc-mir-423-5p and the regulation of ssc-mir-423-5p in the process of spermatogenesis may be mediated by *PLCβ1*.

## Discussion

miRNA regulation is critical and effective mechanism underlying the development of testis and spermatogenesis. The sRNA-seq data in this study elucidated the differences in testis development at different stages between Duroc boars and Meishan boars. The present study obtained approximately 99% clean reads from raw reads in each sample, the percentage of high quality reads has reached nearly 100%. Most of the clean reads (80.56–84.88%) identified in this study could match the *S. scrofa* genome. This result is similar to that found in the study of the pig muscle and ovary transcriptome (78.7%) [23], which indicates that our sRNA-seq data are of high quality. Read length distributions of six libraries demonstrated that 20 to 24 nt represented the length of most sRNAs, of which, 22-nt accounting for the highest percentages. This finding is consistent with the normal size of miRNAs reported in previous study [24]. The present study also indicates that miRNAs are abundantly expressed in testes before puberty, which is consistent with the result that the immature testis had a higher expression level of miRNA than the mature testis [25]. piRNAs are most abundantly expressed in male germ cells, especially during spermatogenesis and these piRNAs are reported to be abundantly expressed in cells at prophase of meiosis I and to get lost at some point before the production of mature sperm [22, 25–27]. piRNAs were abundantly expressed in 75-day-old Meishan boars and 270-day-old Duroc boars, both of which reached puberty and begun to produce sperms [17], while lowly expressed in immature testes (D20, D75, and M20). However, lower expression of piRNAs were also detected in 270-day-old Meishan boars at adulthood than in 75-day-old Meishan boars and 270-day-old Duroc boars, indicating that piRNAs may be the crucial factors causing the differences in sexual function development between Meishan and Duroc boars.

Many mammalian miRNAs play an important role in development and other processes and the expression patterns of miRNAs are tissue-specific or developmental stage-specific [28]. Bioinformatics analyses of miRNAs deriving from the four pairwise comparisons before and after puberty (D20 vs D270, D75 vs D270, M20 vs M75, and M20 vs M270) showed that M20, D20 and D75 were clustered and that M75, M270 and D270 were clustered together (Fig. 2). These findings are in accordance with our previous mRNA-seq analyses [17]. The expression pattern of miRNAs of M75 was similar to that of M270 and D270, which were in adulthood. These results are consistent with the characteristics of early sexual maturity of Meishan boars reaching their puberty prior to 75 days. A large number of miRNAs were up-regulated in M20, D20, and D75, but down-regulated in M75, M270, and D270. This finding agrees with the analysis result of length distribution that miRNAs were abundantly expressed in testes before puberty. These miRNAs may serve potential roles in regulating testis development, and their specific expressions may induce the differences in sexual maturity and spermatogenesis. A growing number of reports have revealed that miRNAs play important roles in the complex processes of animal testis development and spermatogenesis via their regulations of cell proliferation, apoptosis, and differentiation [29–32].

The miRNAs usually negatively regulate gene expressions by binding specific mRNAs in their 3′-(Untranslated regions) UTRs based on sequence complementation to promote degradation of target mRNAs or to inhibit their translation. Recent studies indicate that miRNAs are also involved in positively-regulating gene transcription by targeting promoter elements [33–35]. Emerging evidence shows that miRNAs are also present in the nucleus in addition to their functions in the cytoplasm. Furthermore, the miRNAs in the nucleus exhibit gene-activation function by activating enhancer RNA (eRNA) expression, altering histone modification [36, 37]. The analysis results of negative or positive miRNA/mRNA pairs were consistent with those of the differential expression analyses of miRNAs and mRNAs, suggesting that Meishan boars have an earlier puberty than Duroc boars and that there was a significant difference in sexual function between two breeds. This study verifies *PLCβ1* to be the target gene of ssc-mir-423-5p (Fig. 5) and this gene was also reported to be involved in regulating fertilization rate and embryo development in mice in previous study [38]. In addition, *PLCβ1* was reported to have mapped to GnRH [39] and Wnt [40] signaling pathways regulating testis development and spermatogenesis and sexual reproduction. *SOX30* predicted to be the target gene of ssc-mir-423-5p was also associated with testis development [41]. Thus, it can be inferred that ssc-mir-423-5p may be a potential regulator in the process of testis development and fertilization. To validate the reliability of the data, we selected important DE miRNAs and DEGs related to reproduction and performed qRT-PCR experiments. The qRT-PCR results matched the sRNA-seq and mRNA-seq data.

## Conclusions

In this study, we obtained comprehensive sRNA-seq data and modelled the miRNA-mRNA regulatory networks related to testis development and spermatogenesis through the integrated analysis of differentially expressed miRNAs and target mRNAs between Duroc and Meishan boars. These complex networks identified in our study may participate in regulating boar fertility. In summary, the present study provides an insight into the functional roles of sRNAs in testis development and offers a useful resource for understanding the differences in sexual function and testicular development caused by the change in miRNAs expression between Meishan and Duroc boars.

## Methods

### Tissue collection

The experimental animals in this study were raised under the same conditions. Three 20-, 75-, and 270-day-old clinically healthy Duroc and Chinese Meishan boars were selected from the Fine Farm of Hua Zhong Agricultural University. Boars were given general anesthesia (Zoletil 50, Virbac Co., France), a combination of tiletamine and zolazepam (5–9 mg/kg, i.m.) and xylazine hydrochloride (1.5–2 mg/kg, i.m.) before sampling. Testis samples were collected by castration and immediately snap-frozen in liquid nitrogen and stored at − 80 °C until RNA extraction. Boars were then fed normally for meat industry.

### Illumina small RNA sequencing analysis

Total RNA was extracted using TRIzol reagent (Invitrogen, Carlsbad, CA, USA) following the manufacturer’s instructions. RNA quality and quantity were measured using a Nanodrop 2000 spectrophotometer (Thermo Fisher Scientific, Waltham, MA, USA). Then the equal amount of RNA from three individuals of the same age and breed were mixed to form 6 RNA pools, named D20, D75, D270, M20, M75, and M270. Sequencing libraries were generated using the TruSeq Small RNA Library Prep Kit (Illumina, San Diego, CA, USA), following the manufacturer’s recommendations. Briefly, the small RNA (sRNA) fragments (18–30 nt) were isolated from RNA pools by polyacrylamide gel electrophoresis (PAGE) and 3′ adaptor (TGGAATTCTCGGGTGCCAAGG) was first ligated to the RNA 3′ ends. Then the 5’end adaptor (GTTCAGAGTTCTACAGTCCGACGATC) was ligated to 5’end of the preparation. T4 RNA Ligase (Takara, Dalian, China) was used in the ligation reaction. The adaptor-ligated sRNA was then converted to cDNA using SuperScript II Reverse Transcriptase (Life Technologies, Carlsbad, CA, USA). The resulting cDNA was amplified on the PCR machine. The purified PCR products were recovered with QIAquick Gel Extraction Kit (Qiagen, Beijing, China) following the manufacturer’s instruction and assessed on an Agilent Technologies 2100 Bioanalyzer (Agilent Technologies, Santa Clara, CA, USA). Each sRNA library was sequenced individually using Illumina HiSeq™ 2000 platform (BGI, Shenzhen, China) and 50 nt single-end reads were generated. To predict the genes targeted by miRNAs, two target prediction algorithms (Targetscan 5.0 and miRanda 3.3a) were used to identify miRNA binding sites. Finally, the data obtained were combined and the overlaps were calculated. Integrated analysis between mRNA and miRNA libraries was performed using miRTrail bioinformatics tool.

### Quantitative real-time PCR (qRT-PCR) of miRNA and mRNA expression

To validate the sequencing data, miRNAs and mRNAs were reversely transcribed using RevertAid First Strand cDNA Synthesis Kit (Thermo, Wuhan, k1622) in accordance with the manufacturer’s instructions. The qRT-PCR of the miRNAs and mRNAs was performed by using a standard UltraSYBR Mixture (CWBIO, Beijing, China) in the Roche LightCyler 480 system (Roche, Mannheinm, Germany) according to the manufacturer’s instructions. *U6* and *β-actin* were used as endogenous control genes of miRNA and mRNA, respectively. The primer sequences used for the qRT-PCR were listed in Additional file 7: Table S4. At least three independent biological replicates were used for each of the miRNAs and mRNA. The qRT-PCR data were analyzed by using the 2^-∆∆CT^ method, as previously described [42].

### GO annotation and the KEGG pathway

To further investigate the biological processes and functions which DE miRNAs are involved in through the nine pairwise comparisons, we conducted GO and KEGG pathway analyses. GO enrichment analysis was performed with software Blast2GO, and genes were classified in terms of cellular component, molecular function and biological process using GO annotation. Pathway enrichment analysis was based on KEGG database (http://www.genome.jp/kegg/) and *P* value was used to determine the threshold of significance in multiple tests and analyses with *P* value < 0.05 defined as significant enrichment.

### Plasmid construction

The porcine *PLCβ1* 3’UTR was amplified with primers *PLCβ1*–3’UTR-F/*PLCβ1*–3’UTR-R in Additional file 8: Table S5, and their products were double-digested with *Sac* I (Takara, Dalian, China) and *Xho* I (Takara, Dalian, China), and then was cloned into the pmirGLO vector (Promega, Madison, WI, USA) as a wild-type plasmid. And the wild-type plasmid was used as template to construct mutated plasmid with primers *PLCβ1*–3’UTR-F/*PLCβ1*–3’UTR-MR in Additional file 8: Table S5, and the primer *PLCβ1*–3’UTR-R contained the binding site of ssc-mir-423-5p.

### Cell culture, transfection and dual-luciferase reporter assays

Swine testis (ST) cells (ATCC CRL-1746, Shanghai, China) were cultured with Dulbecco’s modified Eagle medium (DMEM) (Hyclone, Logan, UT, USA) containing 10% fetal bovine serum (CLARK, Worcester, MA, USA) at 37 °C in a humidified atmosphere with 5% CO_2_. The cells were plated into 24 wells. When these cells grew until they reached 70–80% confluent, it was time of transfection. miRNAs were co-transfected with plasmids into the cells using lipofectamine 2000 (Invitrogen, Carlsbad, CA, USA). For luciferase assays, wild-type or mutated plasmids at 200 ng together with 3 μL/well of miRNA mimic, NC, miRNA inhibitor or inhibitor NC (Genepharma, Shanghai, China) were transfected into the cells. Twenty-four hours after transfection, the luciferase activities were measured with a PerkinElmer 2030 Multilabel Reader (PerkinElmer, Waltham, MA, USA).

### Western blotting analysis

Cellular protein lysates were generated using RIPA Lysis Buffer (Beyotime, Jiangsu, China). Cellular proteins were extracted 72 h after transfection. Proteins were heated to 99 °C for 10 min in 5 × SDS buffer, separated by SDS-PAGE, and a Mini Trans-Blot Cell (Bio-Rad, Hercules, CA, USA) was used to transfer protein onto polyvinylidene fluoride membranes (Millipore, Billerica, MA, USA), which were cut to appropriate size according to a protein marker. Then the cut membranes were blocked with 5% non-fat dried milk and incubated overnight with primary antibodies specific for PLCβ1 (1:1000; ABclonal, USA, A1971). β-actin (1:1000; Wuhan, Hubei, China, GB13001–1) used as a loading control. An Image Quant LAS4000 mini (GE Healthcare Life Sciences, Piscataway, NJ, USA) was used to detect protein expression.

### Graphics and heatmaps

A heatmap of miRNAs expression was generated using R heatmap package. All the histograms and graphs were generated with GraphPad Prism and Adobe Photoshop CS5 software, respectively. Molecular interaction network was analyzed by Cytoscape_3.3.0 [43].

### Statistical analysis

All experiments are performed at least in triplicate and the corresponding results are presented in terms of the mean ± SD. Two-tailed t-test was used to reveal the differences between two groups. Significant differences were evaluated through an independent-sample t-test. *P*-value < 0.05 (*P < 0.05; **P < 0.01; ***P < 0.001) was considered to be significant.

## Supplementary information

**Additional file 1: Table S1.** The number of genes detected in the six samples.

**Additional file 2: Table S2.** The number of genes involved in KEGG pathway and GO term.

**Additional file 3: Figure S1.** Expression analyses of 4 DE miRNAs and 4 DEGs using qRT-PCR and RNA-Seq. Analyses of relative expression levels of miRNAs and genes in Duroc (black bars) and Meishan (gray bars) boars in the left; RPKM of miRNAs and genes in Duroc (blue bars) and Meishan (purple bars) boars a in the right. The X-axis indicates age of samples. The left Y-axis and right Y-axis show the relative expression level and RPKM of miRNAs and genes, respectively. a ssc-mir-181b, b ssc-mir-423-5p, c ssc-mir-196b-5p, d ssc-mir-4334-3p, e *CYLD*, f *CDYL*, g *PLCβ1*, h *SOX30*. **P < 0.01, *P < 0.05. DE miRNAs, differentially expressed miRNAs; DEGs, differentially expressed genes; qRT-PCR, quantitative real-time PCR; RNA-Seq, RNA sequencing; RPKM, Reads Per Kilobase per Million mapped reads; *CYLD*, Cylindromatosis; *CDYL*, chromodomain Y like; *PLCβ1*, phospholipase C beta 1; *SOX30*, SRY-box containing gene 30.

**Additional file 4: Table S3.** Synthesis of ssc-mir-423-5p sequences. NC, negative control.

**Additional file 5: Figure S2.** The expression levels of ssc-mir-423-5p were analyzed by qRT-PCR. a ssc-mir-423-5p expression level after transfecting ssc-mir-423-5p mimic, mimic NC. b ssc-mir-423-5p expression level after transfecting ssc-mir-423-5p inhibitor or inhibitor NC into ST cells. NC, negative control; ST, swine testis.

**Additional file 6: Figure S3.** Western blotting analysis was used to detect PLCβ1 protein expression levels at 72 h after ST cells were transfected with ssc-mir-423-5p mimic /mimic NC or ssc-mir-423-5p inhibitor /inhibitor NC.

**Additional file 7: Table S4.** Primers of genes and miRNAs used for quantitative real-time PCR.

**Additional file 8: Table S5.** Primers for amplification of porcine *PLCβ1* 3’UTR.

**Additional file 9: Table S6.** The identified miRNA information.

## Data Availability

All raw data used in this stauy have been submitted to the Sequence Read Archive (https://www.ncbi.nlm.nih.gov/sra/) under accession PRJNA657937.

## Abbreviations

DEGs: Differentially expressed genes
DE miRNAs: Differentially expressed miRNAs
GO: Gene Ontology
KEGG: Kyoto Encyclopedia of Genes and Genomes
miRNAs: MicroRNAs
NC: Negative control
PLCβ1: Phospholipase C beta 1
piRNA: Piwi-interacting RNA
qRT-PCR: Quantitative real-time PCR
sRNA: Small RNA
ST: Swine testis
UTR: Untranslated Region
