# Supplementary material for: Integrated analysis of miRNA and mRNA expression profiles in testes of Duroc and Meishan boars
Source: BMC Genomics. 2020 Oct 2;21:686. doi: 10.1186/s12864-020-07096-7 (PMC7531090; doi:10.1186/s12864-020-07096-7)
Supplement: Supplementary file 1 — Additional file 1: Table S1. The number of genes detected in the six samples. [file 12864_2020_7096_MOESM1_ESM.pdf]

Table S1

Samples	all	D20	D75	D270	M20	M75	M270
Gene number	20,525	18,568	18,510	18,760	18,731	19,015	18,734
Proportion	100%	90.46%	90.18%	91.40%	91.26%	92.64%	91.27%
