# Supplementary material for: Integrated analysis of miRNA and mRNA expression profiles in testes of Duroc and Meishan boars
Source: BMC Genomics. 2020 Oct 2;21:686. doi: 10.1186/s12864-020-07096-7 (PMC7531090; doi:10.1186/s12864-020-07096-7)
Supplement: Supplementary file 2 — Additional file 2: Table S2. The number of genes involved in KEGG pathway and GO term. [file 12864_2020_7096_MOESM2_ESM.pdf]

Table S2

Gene number	KEGG	GO
20525	19310	18241
100%	94.08%	88.87%
