# Supplementary figures and images for: Integrated analysis of miRNA and mRNA expression profiles in testes of Duroc and Meishan boars
Source: BMC Genomics. 2020 Oct 2;21:686. doi: 10.1186/s12864-020-07096-7 (PMC7531090; doi:10.1186/s12864-020-07096-7)

**Figure S1**

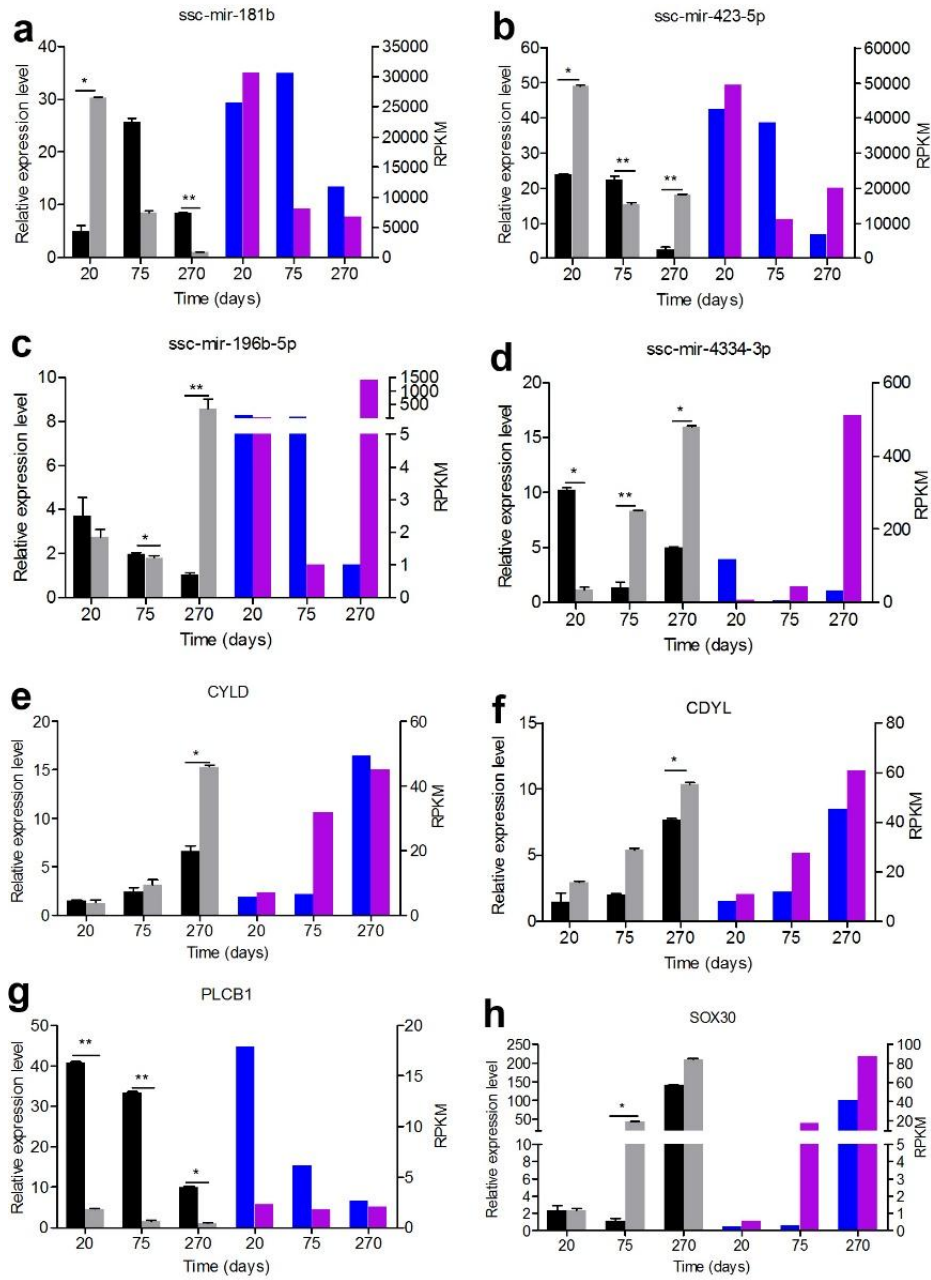

Supplement: Supplementary file 3 — Additional file 3: Figure S1. Expression analyses of 4 DE miRNAs and 4 DEGs using qRT-PCR and RNA-Seq. Analyses of relative expression levels of miRNAs and genes in Duroc (black bars) and Meishan (gray bars) boars in the left; RPKM of miRNAs and genes in Duroc (blue bars) and Meishan (purple bars) boars a in the right. The X-axis indicates age of samples. The left Y-axis and right Y-axis show the relative expression level and RPKM of miRNAs and genes, respectively. a ssc-mir-181b, b ssc-mir-423-5p, c ssc-mir-196b-5p, d ssc-mir-4334-3p, e CYLD, f CDYL, g PLCβ1, h SOX30. **P < 0.01, *P < 0.05. DE miRNAs, differentially expressed miRNAs; DEGs, differentially expressed genes; qRT-PCR, quantitative real-time PCR; RNA-Seq, RNA sequencing; RPKM, Reads Per Kilobase per Million mapped reads; CYLD, Cylindromatosis; CDYL, chromodomain Y like; PLCβ1, phospholipase C beta 1; SOX30, SRY-box containing gene 30. [file 12864_2020_7096_MOESM3_ESM.pdf]

**Figure S2**

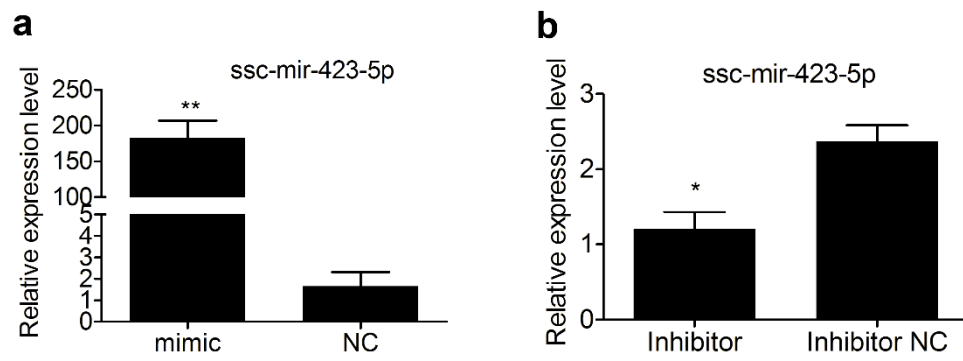

Supplement: Supplementary file 5 — Additional file 5: Figure S2. The expression levels of ssc-mir-423-5p were analyzed by qRT-PCR. a ssc-mir-423-5p expression level after transfecting ssc-mir-423-5p mimic, mimic NC. b ssc-mir-423-5p expression level after transfecting ssc-mir-423-5p inhibitor or inhibitor NC into ST cells. NC, negative control; ST, swine testis. [file 12864_2020_7096_MOESM5_ESM.pdf]
