# Supplementary material for: Integrated analysis of miRNA and mRNA expression profiles in testes of Duroc and Meishan boars
Source: BMC Genomics. 2020 Oct 2;21:686. doi: 10.1186/s12864-020-07096-7 (PMC7531090; doi:10.1186/s12864-020-07096-7)
Supplement: Supplementary file 4 — Additional file 4: Table S3. Synthesis of ssc-mir-423-5p sequences. NC, negative control. [file 12864_2020_7096_MOESM4_ESM.pdf]

Table S3

Sequence name	Sequence (5´-3´)
ssc-mir-423-5p mimic	UGAGGGGCAGAGAGCGAGACUUU<br>AGUCUCGCUCUCUGCCCCUCAUU
Negative control (NC)	UUCUCCGAACGUGUCACGUTT<br>ACGUGACACGUUCGGAGAATT
ssc-mir-423-5p inhibitor	AAAGUCUCGCUCUCUGCCCCUCA
Inhibitor NC	CAGUACUUUUGUGUAGUACAA
