# Supplementary material for: Integrated analysis of miRNA and mRNA expression profiles in testes of Duroc and Meishan boars
Source: BMC Genomics. 2020 Oct 2;21:686. doi: 10.1186/s12864-020-07096-7 (PMC7531090; doi:10.1186/s12864-020-07096-7)
Supplement: Supplementary file 6 — Additional file 6: Figure S3. Western blotting analysis was used to detect PLCβ1 protein expression levels at 72 h after ST cells were transfected with ssc-mir-423-5p mimic /mimic NC or ssc-mir-423-5p inhibitor /inhibitor NC. [file 12864_2020_7096_MOESM6_ESM.docx]

Figure S3

ssc-mir-423-5p inhibitor

inhibitor NC

ssc-mir-423-5p mimic

mimic NC


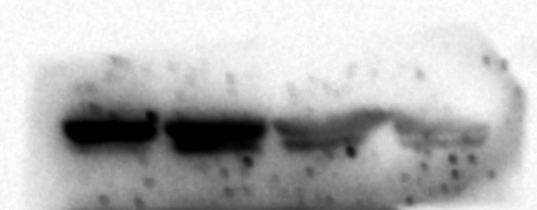


PLCβ1

ssc-mir-423-5p inhibitor

ssc-mir-423-5p mimic

mimic NC

inhibitor NC


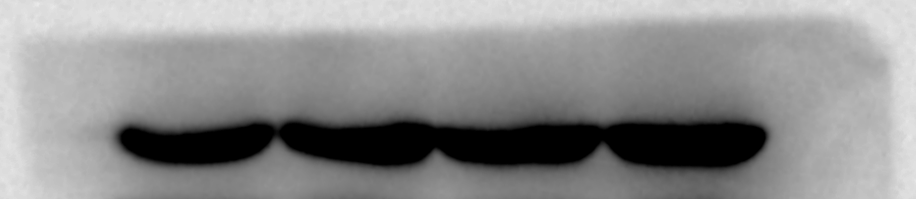


β-actin
