# Supplementary material for: Integrated analysis of miRNA and mRNA expression profiles in testes of Duroc and Meishan boars
Source: BMC Genomics. 2020 Oct 2;21:686. doi: 10.1186/s12864-020-07096-7 (PMC7531090; doi:10.1186/s12864-020-07096-7)
Supplement: Supplementary file 7 — Additional file 7: Table S4. Primers of genes and miRNAs used for quantitative real-time PCR. [file 12864_2020_7096_MOESM7_ESM.pdf]

Table S4

Gene name	Forward sequence (5´-3´)	Reverse sequence (5´-3´)
PLCβ1	AACGAAATACTTTACCCACCC	TCTCCACTCAGATAGCGCATA
SOX30	GGAGTCATCAAAACGGAAGAGC	TGGGTGGAGGTCCCTGAAAT
CDYL	CGGCTTCACCCACATCCT	CCGCCATCTTCGTGCTCT
CYLD	GGCAACACTCAAGTCCACCT	CATTCTGACCACCATCCCG
LDHC	TCTGCCCGTTTCCGTTAC	GACAAGGACCAGTGGAAACG
β-actin	CCAGGTCATCACCATCGG	CCGTGTTGGCGTAGAGGT
scc-mir-196b-5p	ACACTCCAGCTGGGTAGGTAGTTTCCTG	TCAACTGGTGTCGTGGAGTCGGC
ssc-mir-4334-3p	ACACTCCAGCTGGGTCCCTGTCCTCC	TCAACTGGTGTCGTGGAGTCGGC
ssc-mir-423-5p	ACACTCCAGCTGGGTGAGGGGCAGAGAGC	TCAACTGGTGTCGTGGAGTCGGC
ssc-mir-181b	ACACTCCAGCTGGGAAACATTCATTGCTGTC	TCAACTGGTGTCGTGGAGTCGGC
U6	GCTTCGGCAGCACATATACT	TTCACGAATTTGCGTGTCAT
