# Supplementary material for: Integrated analysis of miRNA and mRNA expression profiles in testes of Duroc and Meishan boars
Source: BMC Genomics. 2020 Oct 2;21:686. doi: 10.1186/s12864-020-07096-7 (PMC7531090; doi:10.1186/s12864-020-07096-7)
Supplement: Supplementary file 8 — Additional file 8: Table S5. Primers for amplification of porcine PLCβ1 3’UTR. [file 12864_2020_7096_MOESM8_ESM.pdf]

Table S5

Primer names	Primer sequences (5´-3´)
PLCβ1-3´ UTR-F[1]	CGAGCTC[3]GCGTGGGACTTGGGTTTTCTT
PLCβ1-3´ UTR-R[2]	CCGCTCGAGACAGTTGAGGGGCAGTTAGGG
PLCβ1-3´ UTR-MR	CCGCTCGAGACAGTTGTCCC[4]GCAGTTAGGG

[1] F: forward primer

[2] R: reverse primer

[3] letters with underline: restriction enzyme cutting site

[4] Letters with gray: mutated site
